# Identification of the Tetrel Bonds between Halide Anions and Carbon Atom of Methyl Groups Using Electronic Criterion

**DOI:** 10.3390/molecules24061083

**Published:** 2019-03-19

**Authors:** Ekaterina Bartashevich, Yury Matveychuk, Vladimir Tsirelson

**Affiliations:** 1 Research Laboratory of Multiscale Modelling of Multicomponent Functional Materials, REC Nanotechnology, South Ural State University, 454080 Chelyabinsk, Russia; matveichukyv@susu.ru (Y.M.); vtsirelson@yandex.ru (V.T.); 2 Quantum Chemistry Department, D.I. Mendeleev University of Chemical Technology, 125047 Moscow, Russia

**Keywords:** tetrel bond, electron density, electrostatic potential, potential acting on an electron in molecule

## Abstract

The consideration of the disposition of minima of electron density and electrostatic potential along the line between non-covalently bound atoms in systems with Hal^−^···CH_3_–Y (Hal^−^ = Cl, Br; Y = N, O) fragments allowed to prove that the carbon atom in methyl group serves as an electrophilic site provider. These interactions between halide anion and carbon in methyl group can be categorized as the typical tetrel bonds. Statistics of geometrical parameters for such tetrel bonds in CSD is analyzed. It is established that the binding energy in molecular complexes with tetrel bonds correlate with the potential acting on an electron in molecule (PAEM). The PAEM barriers for tetrel bonds show a similar behavior for both sets of complexes with Br^−^ and Cl^−^ electron donors.

## 1. Introduction

The problem of categorizing non-covalent interactions in molecular crystals and complexes is now a focus of attention [1,2]. Nowadays, the systematization of the halogen, chalcogen, pnictogen, and tetrel bonds already exists [3]; however, in most cases, only the simplistic geometrical approach underlies the analysis of such types of interactions. In this context, the types of non-covalent interactions are traditionally discussed in terms of interatomic distances and angles, which specify the mutual orientation of pivotal chemical bonds [4,5]. However, due to the pronounced and specific electrostatic component of such non-covalent bonds [6,7], more careful analysis of the electronic features of the halogen, chalcogen, pnictogen, and tetrel bonds is required. Such analysis needs to focus on the features of valence electron shells and related anisotropy of the electrostatic potential of interacting atoms.

The estimation of the binding energy for molecules in the Y_4_T···Hal^−^ complexes, where a tetrel atom T = C, Si, Ge, Sn, as well as the description of electron density characteristics for tetrel bonds, were presented in References [8,9,10,11,12,13]. The carbon atom in the СH_3_-group is fairly often noted as the owner of σ-hole, and the fact that the oxygen atom can act as an electron-rich center in the CH_3_···O tetrel bonding has been confirmed in studies [14,15]. Note that in these early works such non-covalent interaction has been referred as a “carbon bond”. Pal et al. [16] described the CH_3_···N tetrel bonding in a Co(II) coordination polymeric system using the analysis of calculated electron density. The typical tetrel bonds formed by СH_3_-group in crystals have been also been observed by high-precision X-ray diffraction method using the analysis of the experimental electron density [17,18].

The rows of binding energy were calculated for the series of complexes in which the compounds NH_3_ [19,20,21,22], PH_3_ and AsH_3_ [23], benzene and unsaturated hydrocarbons [24], HCN and pyrazine [25] acted as the tetrel bond acceptors. According to these results, the tetrel bond strength depends on the tetrel atom, the Lewis base that acts as a tetrel bond acceptor, and the fragments covalently bound with this atom. Therefore, in the complexes with ammonia at transition from CF_4_ to SnF_4_, the binding energy increases from −0.82 to −25.53 kcal/mol (MP2/aug-cc-pVDZ) [21], that is, the tetrels of higher periods form stronger interactions in complexes. Decrease of the Lewis base strength in series from X = NH_3_ to AsH_3_ reduces the binding energy from −3.23 to −1.28 kcal/mol (MP2/aug-cc-pVDZ) in complexes X···CH_3_Cl [23]. Therefore, the values of binding energy estimated in neutral complexes with tetrel bonds formed by the carbon in a methyl group are commonly very small. The binding energy grows more sharply if the hydrogen atoms are replaced by halogens, which leads to enhancement of electron acceptor properties of the tetrel atom providing the σ-hole for bonding. This fact is illustrated by complexes H_4_Sn···NH_3_ (−2.44 kcal/mol) and F_4_Sn···NH_3_ (−25.53 kcal/mol) with the tetrel bond lengths of 3.17 and 2.28 Å (MP2/aug-cc-pVDZ), respectively [21]. If the anion acts as the tetrel bond acceptor, the estimated binding energy can be greater by an order of magnitude as compared to the neutral complexes [9,10,11]. The highest value of binding energy, −93.58 kcal/mol, has been recorded for the SnF_4_···F^−^ complex (MP2/aug-ccpVTZ) [11]; replacing the F^−^ ion with Cl^−^ and Br^−^ reduces this energy by 2–3 times.

Therefore, a variety of functional groups can act as the tetrel bond donors in molecular complexes and a methyl group as an electrophilic site deliverer is quite often in the focus of attention for the study of tetrel bond properties. The tetrel bonds formed by the methyl carbon atom occur in crystalline systems as well. There are at least two studies [17,26] based on high-precision X-ray diffraction data, which established the participation of the carbon atom of a methyl group in non-covalent interactions. These facts motivate us to focus on the search for evidential electronic criterion for recognizing the type of non-covalent bonding and for systematization of the electrophilic site features for the carbon atom of a methyl group in molecular complexes and crystals.

Quantum Theory of Atoms in Molecules (QTAIM) [27] suggests zero-flux conditions for electron-density gradient [28] and electrostatic-potential gradient [29] to determine the boundaries of chemically bonded atoms and electrically neutral atomic fragments, respectively. For non-covalent interactions with a significant electrostatic component, the boundaries of the electron density basins (ρ-basins) do not coincide with the boundaries of the electrostatic potential (ESP) basins (φ-basins). It means that electrons formally belonging to the electron donor atom can be electrostatically attracted to the nucleus of an electron acceptor atom along a specific direction. The superposition of the ρ- and φ-basins has been discussed in the literature [30,31,32], and the features of zero-flux surface in electrostatic potential in solids have been studied using the experimental charge density [29,33].

Previously we have proposed the following electronic criterion for recognizing the atom that prescribes the name of non-covalent bonding [34,35]. The minimum of electrostatic potential along the interaction line is located at the side of the atom that donates electrons; the minimum of electron density is closer to the atom that delivers its electrophilic site for bonding. More explicitly, the latter atom prescribes the name of bonding. This observation opens up the broad possibilities for identifying the role of atoms involved in non-covalent interactions. For example, atoms of the Group 14 of the Periodic Table are able to deliver their electrophilic sites for non-covalent interactions with electron donors for the tetrel bond formation, and the suggested electronic criterion can specify the electron acceptor role of these atoms. Along the Hal^−^···C interatomic line, the 1D minimum of ESP should be located closer to the electron donor atom, while the minimum of the electron density will be found closer to the atom that is an electron acceptor. Such disposition of minima indicates that the fraction of electron density from the atomic ρ-basin of an electron donor is electrostatically attracted to the nucleus of an electron acceptor atom. In this case, the disposition of the 1D minima of both functions can unambiguously indicate that only one of the pair of atoms provides its electrophilic site. Namely, the minimum of electron density on the interatomic line is always closer to the atom that has delivered its own electrophilic site for bonding.

We have recently proposed the potential acting on an electron in molecule (PAEM [36]) [37] as a function that not only characterizes the properties of non-covalent bonding with a significant electrostatic component, but also allows us to observe the quantitative relationship with the interaction energy in complexes. Unlike ESP, the PAEM contains both Coulomb and exchange components. The first of them has a classic nature and the second one is the two-electron contribution of the quantum exchange-correlation potential. PAEM was examined [38] for the halogen and chalcogen bonding characterization and its usefulness was confirmed.

The aim of the present study is to demonstrate the efficiency and productivity of the above-mentioned electronic criterion and the PAEM for analysis of tetrel bonds between the carbon atom of methyl groups and halide anions, which sometimes occur in molecular crystals. We also try to understand to what extent the characteristics of the gas-phase complexes are suitable for describing Hal^−^···CH_3_Y (Hal^−^ = Cl, Br; Y = N, O) tetrel bonds in crystals.

## 2. Results and Discussion

### 2.1. Population of Hal^−^···CH_3_Y Tetrel Bonds in Crystals

**Figure 1:**
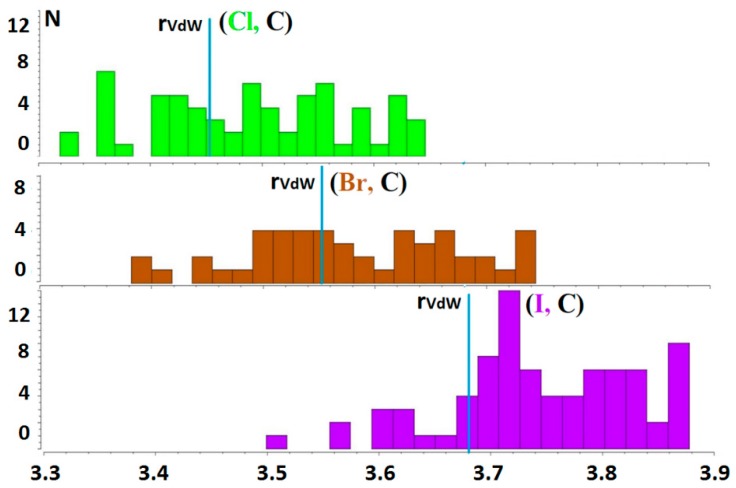
The distribution of the Hal^−^···CH_3_–Y interactions in crystals for Cl^−^ (green), Br^−^ (brown), I^−^ (violet); the blue lines mark the sum of van der Waals radii.

The search of short contacts between a halide anion and the methyl carbon, Hal^−^···CH_3_–Y, in Cambridge Structural Database (CSD) v.5.39 (The Cambridge Crystallographic Data Centre, Cambridge, UK) [39] was performed with the following restrictions: organic derivatives without disordered, polymeric, powder, organometallic and repeating structures have been considered. The main condition was to choose interactions in which the halide anion, Hal^−^ = Cl, Br, I, was placed on the extension of the C–Y covalent bond, where Y = C, N, O. We set that condition using the angle θ (Hal^−^–C–Y), the value of which was in the range from 160° to 180°. At the same time, we selected the structures with interatomic distances, d(Hal, C), falling into the range (r_vdw_(C) + r_vdw_(Hal) ± 0.2 Å), where r_vdw_ is the Bondi atomic radius [40]. The total number of selected structures that satisfied those conditions was 164. The analysis of the obtained sample has shown that the Y atom covalently bound with the CH_3_ group is nitrogen in most cases. We have found 43 cases of Cl^−^···CH_3_–N interactions, 36 cases of Br^−^···CH_3_–N interactions and 53 cases of I^−^···CH_3_–N interactions. Oxygen and carbon are involved in such covalent bonds much less frequently and approximately equally. In these cases, the methyl group forms more interactions with the Cl^−^ anion than with the Br^−^ and I^−^ anions taken together. It can be concluded that the polarity of the covalent bond CH_3_–Y affects the probability and strength of the tetrel bond formation. It should be noted that the distances d(Hal, C) for the cases of Hal^−^···CH_3_–C interactions everywhere exceed the sum of van der Waals radii (Figure 1). If the CH_3_-group is bound with oxygen (Hal^−^···CH_3_–O), the distances d(Hal, C) can be less than the sum of van der Waals radii, though all of these distances are more than this sum for I^−^ cases.

Therefore, we conclude that the studied type of interactions, Hal^−^···CH_3_–Y, are not widely spread within crystals listed in CSD, but they are not exceptional.

### 2.2. Evidence of Electrophilic Sites for the CH_3_-Groups Bound in Tetrel Bonds

**Figure 2:**
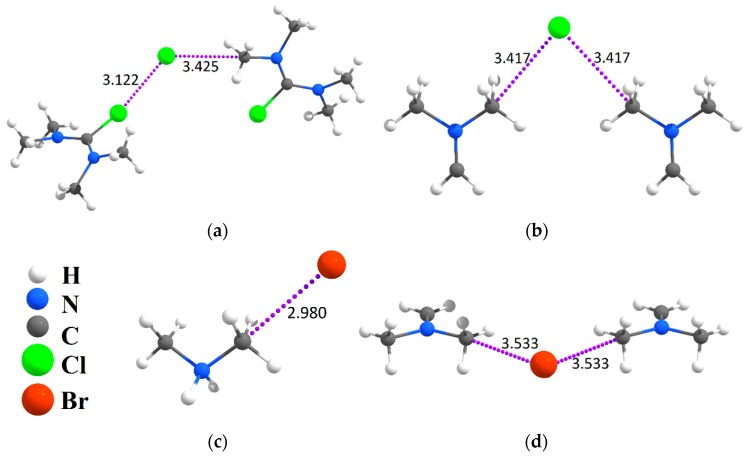
Fragments of structures with tetrel bonds and other non-covalent interactions in crystals LONGEB (**a**), VAPREJ (**b**), POSTUM02 (**c**), LILLOH (**d**). The interatomic distances are given in Angstroms.

Let us now look at the same examples of halide crystal structures containing tetrel bonds (Figure 2). All the results considered in this chapter were obtained for crystal structures by the calculations with the periodic boundary conditions. In the crystalline *N*,*N*,*N*′,*N*′-tetramethylchloroformamide chloride, LONGEB [41], the Cl^−^ anion forms non-covalent interactions with the Cl, H, C atoms, which are characterized by interatomic distances smaller than the sums of van der Waals radii. The Cl^−^···Cl–C non-covalent interaction with a distance of 3.122 Å refers to a typical charge-assisted halogen bond; the next five hydrogen bonds, Сl^−^···H–C, are characterized by interatomic distances ranging from 2.924 to 2.664 Å. Finally, the Cl^−^···CH_3_ interaction of 3.425 Å can be called a tetrel bond. In the crystalline dimethylmethyleneammonium chloride VAPREJ [42], the chloride anion forms eight Сl^−^···H–C interactions, which are shorter than the sum of van der Waals radii and two Cl^−^···CH_3_ tetrel bonds. In the dimethylmethylenimine bromide crystal, LILLOH [43], in addition to multiple Br^−^···H–C interactions, there are two tetrel bonds: Br^−^···CH_3_ (3.533 Å) and Br^−^···CH_2_ (3.503 Å). Quantum-topological analysis of the electron density in all considered crystals have confirmed the presence of the Hal^−^···C bond path and bcp of electron density (Table 1). Our series of tetrel bonds in the considered crystals does not vary much, and we have observed the small changes in electron density at the bond critical points, ρ(r_bcp_), which are in the range 0.0042–0.0070 a.u. for Cl^−^···CH_3_–Y and 0.0060–0.0068 a.u. for Br^−^···CH_3_–Y.

**Figure 3:**
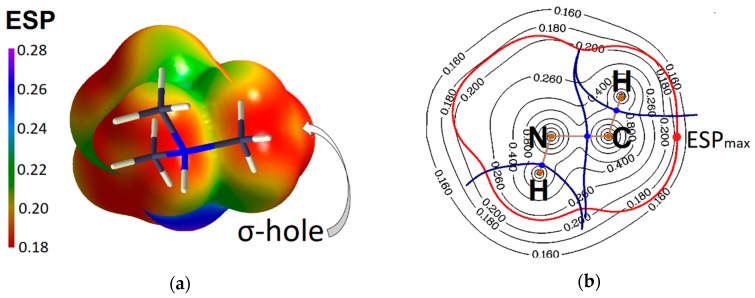
(**a**) ESP in the trimethylammonium cation on the isosurface of electron density of 0.003 a.u.; (**b**) contour map of ESP in the plane N-C-H, red point indicates the maximum of ESP on the van der Waals surface (red line) and belongs to C atomic basin.

It is possible to demonstrate the electrophilic site on a carbon atom using the electrostatic potential (ESP) mapped on the isosurface of electron density or the distribution of Electron Localization Function (ELF) [44] for CH_3_-group, which participate in a tetrel bond. For example, relatively higher positive values of ESP on the isosurface of electron density (0.003 a.u.) we can see in the region of the σ-hole, which belongs to the C atomic basin in trimethylammonium cation (Figure 3a,b).

**Figure 4:**
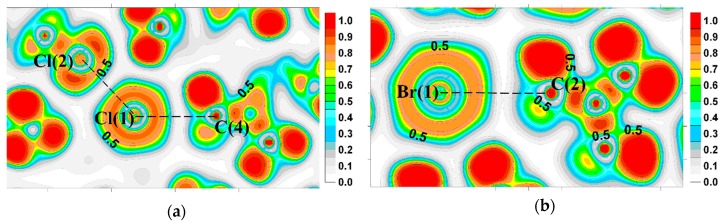
The ELF for (**a**) the halogen and tetrel bonds Cl(2)···Cl(1)^−^···C(4) in LONGEB crystal; (**b**) the tetrel bond in LILLOH crystal.

Moving strictly along the line linking the Сl(1)^−^ and C(4) atoms of CH_3_-group (Figure 4а), we reach the extension of the covalent bond formed by CH_3_-group. Along this line the ELF is less than 0.5 near the carbon atom, showing the region of the reduced probability of electron pairing. It can be considered as the manifestation of the carbon atom σ-hole. The values of ELF (r_bcp_) at the bond critical points do not exceed 0.05. This excludes the hypothesis about significant covalent character of the Hal^−^···CH_3_Y tetrel bonds. At about 0.8 Å from the chloride anion nucleus the maximum values of ELF are distributed around the circumference. Figure 4b, depicting the tetrel bonds formed by the bromide anion in LILLOH crystal, shows a similar ELF distribution. Near the carbon atom and along the Br(1)^−^ ···C(2) line the ELF does not attain high values, but it increases sharply, affecting the basins of hydrogen atoms, if we slightly deviate from this line.

**Figure 5:**
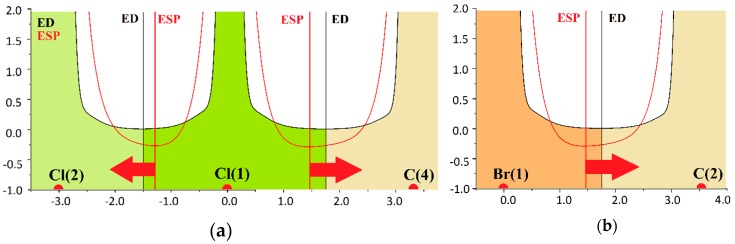
The disposition of electron density and electrostatic potential minima (a.u.) (**a**) along two interatomic lines Cl(2)···Cl(1)^−^···C(4), (Å), in LONGEB; (**b**) along the tetrel bond in LILLOH. The arrows point to the electrophilic site provider.

Now let us consider and evaluate how the electronic criterion works for the cases of non-covalent interactions formed by the carbon atoms of methyl groups in halide crystals. In Figure 5a it can be seen that in the LONGEB crystal, the Cl^−^ anion forms two non-covalent interactions at least, as follows from the presence of corresponding bcp. In both cases, Cl(2)···Cl(1)^−^···C(4), the one-dimensional ESP minimum is closer to electron donating anion, Cl(1)^−^ in a crystal. The electron density minima along the Cl(2)···Cl(1)^−^ and Cl(1)^−^···C(4) lines are located on the side of the Cl(2) and C(4) atoms. They indicate the electrophilic site providers and dictate the name of the non-covalent bonding.

According to the proposed electronic criterion, the first interaction, Cl(2)···Cl(1)^−^, can be categorized as a charge-assisted halogen bond, and the second one, Cl(1)^−^···C (4), is a tetrel bond enhanced by charges. In Figure 5b, the minimum of electron density along the Br(1)^−^···C(2) line is located on the side of the C(4) atom, while the minimum of ESP is closer to Br(1)^−^. Such disposition of minima shows that the carbon atom accepts electrons along the Br(1)^−^···C(2) line and that interaction can be called a tetrel bond.

### 2.3. Binding Energy in Molecular Complexes with the Hal^−^···CH3 Tetrel Bonds

The determination of the equilibrium geometry for ion pairs “halide anion–cation” extracted from the crystal environment is not a straightforward procedure. In general, the retention of the halide anion position strictly on the extension of a covalent bond of CH_3_-group is rather difficult in the gas phase state. This task requires us to dwell on the level of gas phase calculations, different from those used for crystal structures. Nevertheless, this step allows us to obtain the stationary state for the maximal number of complexes, for which the tetrel bonds in crystalline state have attracted our attention. Some relative estimations and the features of electronic properties can be quite useful for understanding the nature of charge-assisted tetrel bonds.

The binding energy, E_b_, between the halide anion and cation in the considered complexes varies from −52.28 to −82.67 kcal/mol (Table 2). These values do not fall out of the range that is determined in similar studies [8,9,10,11]. The BSSE correction, ΔE_BSSE_, is negligible and influences the energy values of the third decimal place of kcal/mol units.

The considered tetrel bonds exhibit significantly shorter lengths in the models of complexes extracted from the crystalline environment. On average, the observed Hal^−^···C bond lengths in such complexes differ by ~17% from those in crystal structures. As a result, the different approaches for complexes and crystals calculations lead to the values of ρ(r_bcp_) that are almost twice higher in crystals. Obviously, the direct transfer of tetrel bond properties in isolated complexes to the crystals, neglecting the rest of interactions between a halide anion and crystalline environment, is not entirely correct. Comparing the properties of the CH_3_–Y (Y=N, O) covalent bonds in complexes and isolated cations, we see that the participation of CH_3_-group in tetrel bond with Hal^−^ weakens the CH_3_–Y covalent bond. Therefore, the tetrel bonding elongates the covalent bond of a methyl group by 0.01–0.04 Å, and the values of ρ(r_bcp_) for the CH_3_–Y bonds decrease by ~8%.

It is useful to understand how the electronic properties of Hal^−^···CH_3_Y tetrel bonds in complexes are related to the strength of complexes. In our opinion, the tetrel bonds belong to electrostatically driven interactions. In addition, the binding energy between two oppositely charged ions is much higher in comparison with neutral molecules. For this reason, the electrostatic properties of tetrel bonds have been analyzed first of all.

**Figure 6:**
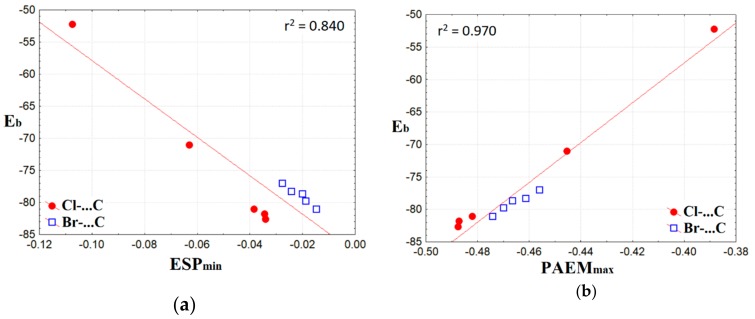
Binding energy in complexes vs minimum of electrostatic potential (**a**) and maximum of potential acting on an electron in a molecule, (**b**) along the line of tetrel bonds.

We found that the properties of both ESP and PAEM for the Hal^−^···CH_3_Y tetrel bonds are linearly correlated with the binding energy, E_b_, in complexes, as shown in Figure 6. The correlation coefficient for the minima of the electrostatic potential, ESP_min_, on the line between Hal^−^ and C atoms is 0.917. For the maximum of PAEM along this line, PAEM_max_, or PAEM barrier, the correlation coefficient is 0.985. It is important to note that in the relationship “PAEM_max_ vs E_b_“, PAEM_max_ for the tetrel bonds formed by Br^−^ and Cl^−^ fits strictly on the common line. This is a rare case among the established relationships between local properties of non-covalent bonds and the binding energy for bound fragments. For example, the electronic potential and kinetic energy densities at bcp do not allow constructing a good common relationship for Br^−^ and Cl^−^ rows (Figure S1). This finding has been discussed by us earlier for the halogen bonds formed by different atoms or fragments that play the role of halogen acceptors [45]. This fact has recently been illustrated in detail in Reference [46], where the large series of non-covalent interactions with different halide anions have been studied.

**Figure 7:**
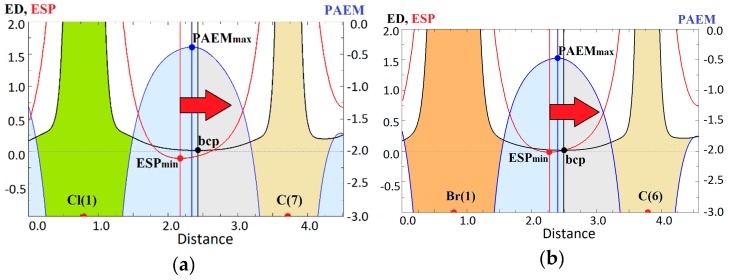
Potential acting on an electron in a molecule, а.u., (blue), electrostatic potential, а.u., (red) and electron density, а.u., (black) along the tetrel bond in (**a**) ZENJAD and (**b**) FADXIR complexes.

Note that the extreme values of ESP_min_ and PAEM_max_ slightly differ from their local values at the bond critical points of tetrel bonds (Table S4). Nevertheless, the PAEM(r_bcp_) values correlate with the binding energy better than ESP(r_bcp_) (Figure S2). This is probably due to the fact that in our series the maximum of PAEM is closer to the tetrel bond critical point than the minimum of ESP (Figure 7). The relative location of ESP_min_ and PAEM_max_ in the common projection is demonstrated by examples of the weakest Cl^−^···CH_3_ (ZENJAD) and the strongest Br^−^···CH_3_ (FADXIR) tetrel bonds in our set. Though the gap between the PAEM_max_ and the minimum of electron density is larger for the Cl^−^···CH_3_ tetrel bond, and ESP has a lower negative minimum, it can be seen that the PAEM barrier is higher in absolute value. It means that the Cl^−^···CH_3_ tetrel bond is weaker, and this is confirmed by the linear correlation between PAEM_max_ and E_b_. Moreover, the relative positions of ESP_min_ and PAEM_max_ along the tetrel bond line allow us to distinguish, which atom is the acceptor of electrons. As it has been noted earlier [45], and as can be seen from the above, PAEM_max_ position is located closer to the electrophilic site, while the position of ESP_min_ is closer to the electron donor.

## 3. Materials and Methods

The structure optimization of molecular complexes consisting of organic cations and halide anions was carried out at M06-2X/aug-cc-pVDZ level [47,48,49] in GAMESS (v. 2017 R2, Mark Gordon’s Quantum Theory Group, Ames Laboratory, Iowa State University, Ames, IA, USA [50]) with gradient convergence that equaled 0.00001. The optimized structures were tested for the absence of imaginary frequencies. The binding energy between cations and halide anions in electrically neutral complexes was estimated as E_b_ = E_com_ − (E_Hal_ + E_cat_) − ΔE_BSSE_, where E_com_, E_cat_, E_Hal_ were the total energies of the optimized complex, relaxed isolated organic cation and halide anion. BSSE correction, ΔE_BSSE_, was carried out taking into account the phantom orbitals in complexes calculated for compounds without energy relaxation, see Table S1 in Supporting Information.

All calculations with periodic boundary conditions were performed using CRYSTAL14 (v. 1.0.4, CRYSTAL Theoretical Chemistry Group, Chemistry Department, University of Turin, Turin, Italy [51]) at the B3LYP/6-31G** level for C, N, O, H atoms and DZVP basis set for halogen atoms [52,53] with Grimme dispersion correction D2 [54]. The structure relaxation was carried out with the atomic coordinate optimization only, with the fixed unit cell parameters for the purpose of maximum conformity to experimental data. The following convergence parameters have been used for all calculation: TOLDEG (root-mean-square on gradient) is less than 0.0001 a.u., TOLDEX (root-mean-square on estimated displacements) is less than 0.0003 a.u., TOLDEE (energy change between optimization steps threshold) is less than 10^−10^ a.u., TOLINTEG (truncation criteria for bielectronic integrals: overlap threshold for Coulomb integrals; penetration threshold for Coulomb integrals; overlap threshold for HF exchange integrals; pseudo-overlap for g and n HF exchange series) are 10, 10, 10, 10 and 16, respectively. The number of k-points in the Pack–Monkhorst net (in the irreducible part of Brillouin zone) was 125 or 170 depending on crystals; the number of k-points in the Gilat net was 729 or 1170, that corresponded to the set SHRINK 8 16 values. All calculations for isolated cations were performed using CRYSTAL17 (v. 1.0.2, CRYSTAL Theoretical Chemistry Group, Chemistry Department, University of Turin, Turin, Italy [55]) at the B3LYP/6-31G** level with the Grimme dispersion correction D2 and DOPING option to account for the cation positive charge.

The QTAIM analyses of electron density and electrostatic potential were carried out in TOPOND [56] in crystals and in AIMAll software package [57] for the complexes. PAEM and ESP distributions were computed using Multiwfn [58] program (Beijing Kein Research Center for Natural Sciences, Beijing, China).

The reported calculations were performed using the supercomputer resources of the South Ural State University [59].

## 4. Conclusions

In this computational study, the charge-assisted tetrel bonds in the crystals formed between halide anions and the methyl groups of organic cations such as Hal^−^···CH_3_Y (Hal^−^ = Cl, Br; Y = N, O) have been considered. The bond paths between the Hal^−^ and C atoms confirm the existence of these uncommon bonds in both the crystal structures and gas phase complexes. To define the type of Hal^−^···CH_3_Y bonding in crystals more precisely, we have suggested using the order of one-dimensional minima of electron density and electrostatic potential along the interatomic lines between the carbon atom of СH_3_-group and the halide anion. This allowed us to apply a simple criterion which reveals that the carbon atom provides its electrophilic site for a typical tetrel bond formation.

The strong correlation between the binding energy in complexes and the extreme values of potential acting on an electron in a molecule calculated along the lines between the Hal^−^ and C atoms has been obtained. Therefore, PAEM extends and enforces the electronic criterion for revealing electrophilic sites and sheds some light on the nature of tetrel bonds. We may speculate that its application will be useful for the other electrostatically driven non-covalent interactions as well.

## Figures and Tables

**Table 1:** Experimental and calculated tetrel and C–Y bond lengths, D (Å), angles Hal^−^···C–Y, θ and calculated electron density, ρ(r_bcp_) (a.u.) at bond critical points for considered crystals.

Crystal	Bond	Crystal D_exp_, θ (Hal^−^···C–N)_exp_	Crystal D_calc_, θ (Hal^−^···C–N)_calc_	ρ(r_bcp_), Crystal
GETQIF	Сl(3)^−^ ···C(2)	3.4584 169.08	3.4260 163.91	0.0056
	C(2)–N(1)	1.4815	1.4958	0.2441
LONGEB	Сl(1)^−^ ···C(4)	3.4251 175.28	3.4087 166.64	0.0068
	C(4)–N(2)	1.4722	1.4740	0.2458
VAPREJ	Сl(1)^−^ ···C(1)	3.417 164.88	3.4385 164.49	0.0064
	C(1)–N(1)	1.466	1.4747	0.2480
TMHYZC	Сl(1)^−^ ···C(2)	3.4374 174.96	3.4280 176.34	0.0062
	C(2) ···N(1)	1.4976	1.5080	0.2406
ZENJAD	Сl(1)^−^ ···C(7)	3.5111 170.58	3.4644 171.72	0.0056
	C(7)–O(2)	1.4471	1.4468	0.2306
LILLOH	Br(1)^−^ ···C(2)	3.533 167.25	3.5664 166.86	0.0061
	C(2)–N(1)	1.474	1.4735	0.2490
FADXIR	Br(1)^−^ ···C(6)	3.6014 170.87	3.5722 173.15	0.0058
	C(6)–N(1)	1.5025	1.5082	0.2371
POSTUM02	Br(1)^−^ ···C(1)	3.7012 175.21	3.6667 174.15	0.0048
	C(1)–N(1)	1.4852	1.4928	0.2432
ZZZGVM01	Br(1)^−^ ···C(2)	3.742 168.65	3.7283 169.04	0.0042
	C(2)–N(1)	1.474	1.4968	0.2437
ZZZUQO03	Br(1)^−^ ···C(1)	3.685 171.12	3.6819 171.11	0.0049
	C(1)–N	1.487	1.5039	0.2411

**Table 2:** Calculated binding energy E_b_ (kcal/mol), bond lengths D_calc_ (Å), electron density ρ(r_bcp_) at bcp (a.u.) for Hal^−^···CH_3_ minimum of electrostatic potential ESP_min_ (а.u.) and maximum of potential acting on an electron in molecule PAEM_max_ (а.u.) along the line of tetrel bonds in complexes.

Refcode	Tetrel Bond	E_b_	D_calc_	ρ(r_bcp_)	ESP_min_	PAEM_max_
GETQIF	Сl^−^···CH_3_Y	−81.07	2.8262	0.019	−0.038	−0.4819
LONGEB	Сl^−^···CH_3_Y	−71.05	2.8782	0.017	−0.063	−0.4454
TMHYZC	Сl^−^···CH_3_Y	−82.67	2.8248	0.019	−0.034	−0.4874
VAPREJ	Сl^−^···CH_3_Y	−81.80	2.8226	0.019	−0.034	−0.4870
ZENJAD	Сl^−^···CH_3_Y	−52.28	2.9268	0.015	−0.107	−0.3883
FADXIR	Br^−^···CH_3_Y	−81.09	2.9855	0.017	−0.015	−0.4741
LILLOH	Br^−^···CH_3_Y	−78.67	2.9896	0.016	−0.020	−0.4664
POSTUM02	Br^−^···CH_3_Y	−79.82	2.9802	0.017	−0.019	−0.4699
ZZZGVM01	Br^−^···CH_3_Y	−77.97	2.9949	0.0165	−0.024	−0.4612
ZZZUQO03	Br^−^···CH_3_Y	−76.63	3.0015	0.0162	−0.028	−0.4560

## Supplementary Materials

The following are available online, Figure S1: Binding energy (kcal/mol) in complexes vs the potential (a) and kinetic (b) energy density (a.u.) at the bond critical point of tetrel bonds, Figure S2: Binding energy (kcal/mol) in complexes vs the electrostatic potential (a.u.) (a) and (b), potential acting on an electron in molecule (a.u.) at the bond critical point of tetrel bonds, Figure S3: ESP in the trimethylammonium chloride on the isosurface of electron density of 0.02 a.u, Table S1: The energy characteristics of Hal^−^···CH_3_–YR (Hal^−^ = Cl, Br) complexes taken from crystal structures with listed refcodes, Table S2. Experimental and calculated tetrel and C–Y bond lengths D (Å), angles Hal^−^···C–Y and electron density (a.u.) at bond critical points for considered crystal and cation structures calculated in CRYSTAL code. Table S3, Bond lengths D(Å), the characteristics of electron density, potential and kinetic energy densities (a.u.), electrostatic potential (a.u.), potential acting on an electron in molecule PAEM at bcp (a.u.) for Hal^−^···CH_3_ and Y–C bonds in complexes and cations calculated in GAMESS code.

## References

[1] Legon A.C. (2017). Tetrel, Pnictogen and Chalcogen Bonds Identified in the Gas Phase before they had Names: A Systematic Look at Non-covalent Interactions. Phys. Chem. Chem. Phys..

[2] Cavallo G., Metrangolo P., Pilati T., Resnati G., Terraneo G. (2014). Naming Interactions from the Electrophilic Site. Cryst. Growth Des..

[3] Terraneo G., Resnati G. (2017). Bonding Matters. Cryst. Growth Des..

[4] Desiraju G.R., Parthasarathy R. (1989). The Nature of Halogen···Halogen Interactions: Are Short Halogen Contacts Due to Specific Attractive Forces or Due to Close Packing of Nonspherical Atoms?. J. Am. Chem. Soc..

[5] Desiraju G.R., Ho P.S., Kloo L., Legon A.C., Marquardt R., Metrangolo P., Politzer P., Resnati G., Rissanen K. (2013). Definition of the Halogen Bond (IUPAC Recommendations 2013). Pure Appl. Chem..

[6] Politzer P., Murray J.S., Clark T. (2010). Halogen Bonding: An Electrostatically-Driven Highly Directional Noncovalent Interaction. Phys. Chem. Chem. Phys..

[7] Politzer P., Murray J.S., Clark T., Resnati G. (2017). The σ-hole Revisited. Phys. Chem. Chem. Phys..

[8] Del Bene J.E., Alkorta I., Elguero J. (2016). Anionic Complexes of F^−^ and Cl^−^ with Substituted Methanes: Hydrogen, Halogen, and Tetrel Bonds. Chem. Phys. Lett..

[9] Scheiner S. (2018). Tetrel Bonding as a Vehicle for Strong and Selective Anion Binding. Molecules.

[10] Esrafili M.D., Asadollahi S., Mousavian P. (2018). Anionic Tetrel Bonds: An ab Initio Study. Chem. Phys. Lett..

[11] Esrafili M.D., Mousavian P. (2018). Strong Tetrel Bonds: Theoretical Aspects and Experimental Evidence. Molecules.

[12] Grabowski S.J. (2014). Tetrel bond–σ-hole bond as a preliminary stage of the S_N_2 reaction. Phys. Chem. Chem. Phys..

[13] Mani D., Arunan E. (2013). The X–C···Y (X = O/F, Y = O/S/F/Cl/Br/N/P) ‘carbon bond’ and hydrophobic interactions. Phys. Chem. Chem. Phys..

[14] Bauza A., Frontera A. (2016). RCH_3_···O Interactions in Biological Systems: Are They Trifurcated H-Bonds or Noncovalent Carbon Bonds?. Crystals.

[15] Garcia-LLinas X., Bauza A., Seth S.K., Frontera A. (2017). Importance of R−CF_3_···O Tetrel Bonding Interactions in Biological Systems. J. Phys. Chem. A.

[16] Pal P., Konar S., Lama P., Das K., Bauza A., Frontera A., Mukhopadhyay S. (2016). On the Importance of Noncovalent Carbon Bonding Interactions in the Stabilization of a 1D Co(II) Polymeric Chain as Precursor of a Novel 2D Coordination Polymer. J. Phys. Chem. B.

[17] Tsirelson V.G., Stash A.I., Potemkin V.A., Rykounov A.A., Shutalev A.D., Zhurova E.A., Zhurov V.V., Pinkerton A.A., Gurskaya G.V., Zavodnik V.E. (2006). Molecular and Crystal Properties of Ethyl-4,6-dimethyl-2-thioxo-1,2,3,4-tetrahydropyrimidine-5-carboxylate from Experimental and Theoretical Electron Densities. Acta Cryst. B.

[18] Thomas S.P., Pavan M.S., Row T.N.G. (2014). Experimental Evidence for ‘Carbon Bonding’ in the Solid State from Charge Density Analysis. Chem. Commun..

[19] Scheiner S. (2018). Steric Crowding in Tetrel Bonds. J. Phys. Chem. A.

[20] Zierkiewicz W., Michalczyk1 M., Scheiner S. (2018). Comparison between Tetrel Bonded Complexes Stabilized by σ and π Hole Interactions. Molecules.

[21] Scheiner S. (2017). Systematic Elucidation of Factors That Influence the Strength of Tetrel Bonds. J. Phys. Chem. A.

[22] Liu M., Li Q., Scheiner S. (2017). Comparison of Tetrel Bonds in Neutral and Protonated Complexes of PyridineTF_3_ and FuranTF_3_ (T = C, Si, and Ge) with NH_3_. Phys. Chem. Chem. Phys..

[23] Laconsay C.J., Galbraith J.M. (2017). A Valence Bond Theory Treatment of Tetrel Bonding Interactions. Comp. Theor. Chem..

[24] Grabowski S.J. (2018). Tetrel Bonds with π-Electrons Acting as Lewis Bases—Theoretical Results and Experimental Evidences. Molecules.

[25] Zierkiewicz W., Michalczyk1 M., Scheiner S. (2018). Implications of Monomer Deformation for Tetrel and Pnicogen Bonds. Phys. Chem. Chem. Phys..

[26] Stash A.I., Chen Y.S., Kovalchukova O.V., Tsirelson V.G. (2013). Electron Density, Electrostatic Potential, and Spatial Organization of Ammonium Hydrooxalate Oxalic Acid Dihydrate Heteromolecular Crystal from Data of Diffraction Experiment at 15 K Using Synchrotron Radiation and Theoretical Calculations. Russ. Chem. Bull..

[27] Bader R.F.W. (1990). Atoms in Molecules: A Quantum Theory.

[28] Bader R.F.W., Carroll M.T., Cheeseman J.R., Chang C. (1987). Properties of Atoms in Molecules: Atomic Volumes. J. Am. Chem. Soc..

[29] Tsirelson V.G., Avilov A.S., Lepeshov G.G., Kulygin A.K., Stahn J., Pietsch U., Spence J.C.H. (2001). Quantitative Analysis of the Electrostatic Potential in Rock-Salt Crystals Using Accurate Electron Diffraction Data. J. Phys. Chem..

[30] Tsirelson V.G., Shishkina A.V., Stash A.I., Parsons S. (2009). The Experimental and Theoretical QTAIMC Study of the Atomic and Molecular Interactions in Dinitrogen Tetroxide. Acta Crystallogr. Sect. B Struct. Sci. Cryst. Eng. Mater..

[31] Mata I., Molins E., Alkorta I., Espinosa E. (2007). Topological Properties of the Electrostatic Potential in Weak and Moderate N···H Hydrogen Bonds. J. Phys. Chem..

[32] Bartashevich E.V., Yushina I.D., Kropotina K.K., Muhitdinova S.E., Tsirelson V.G. (2017). Testing the Tools for Revealing and Characterizing the Iodine-Iodine Halogen Bond in Crystals. Acta Crystallogr. Sect. B Struct. Sci. Cryst. Eng. Mater..

[33] Pathak R.K., Gadre S.R. (1990). Maximal and Minimal Characteristics of Molecular Electrostatic Potentials. J. Chem. Phys..

[34] Bartashevich E.V., Yushina I.D., Stash A.I., Tsirelson V.G. (2014). Halogen Bonding and Other Iodine Interactions in Crystals of Dihydrothiazolo(oxazino)quinolinium Oligoiodides from the Electron-Density Viewpoint. Cryst. Growth Des..

[35] Bartashevich E.V., Yushina I.D., Muhitdinova S.E., Tsirelson V.G. (2019). Electronic Criterion for Categorizing the Chalcogen and Halogen Bonds: Sulfur—Iodine Interactions in Crystals. Acta Crystallogr. Sect. B Struct. Sci. Cryst. Eng. Mater..

[36] Zhao D.-X., Gong L.-D., Yang Z.-Z. (2005). The Relations of Bond Length and Force Constant with the Potential Acting on an Electron in a Molecule. J. Phys. Chem. A.

[37] Bartashevich E.V., Tsirelson V.G. (2018). A Comparative View on the Potential Acting on an Electron in a Molecule and the Electrostatic Potential through the Typical Halogen Bonds. J. Comput. Chem..

[38] Bartashevich E.V., Mukhitdinova S.E., Tsirelson V.G. (2018). Characterizing the Halogen and Chalcogen Bonds in Crystals: PAEM vs ESP. Book of Abstracts of International Union of Crystallography (IUCr)’s Sagamore XIX Conference on Quantum Crystallography, Halifax, NS, Canada.

[39] Groom C.R., Bruno I.J., Lightfoot M.P., Ward S.C. (2016). The Cambridge Structural Database. Acta Crystallogr. Sect. B Struct. Sci. Cryst. Eng. Mater..

[40] Bondi A. (1964). Van der Waals Volumes and Radii. J. Phys. Chem..

[41] Tiritiris I., Kantlehner W. (2008). Crystal Structure of *N*,*N*,*N*′,*N*′-tetramethylchloroformamidinium chloride, [C_5_H_12_N_2_Cl]Cl. Z. Kristallogr. New Cryst. Struct..

[42] Burg A.B. (1989). Restudy of the Action of Sulfur Dioxide on Dry Trimethylamine Oxide: Iodine Oxidation and Lewis Acid Chemistry of the Most Reactive Product, (CH_3_)_2_(H)NCH_2_SO_3_. Inorg. Chem..

[43] Clark G.R., Shaw G.L., Surman P.W.J., Taylor M.J., Steele D. (1994). Preparation, Structure and Vibrational Spectrum of the Dimethylmethyleniminium Ion, Including the Role of Cationic Polymers in its Formation. J. Chem. Soc. Faraday Trans..

[44] Silvi B., Savin A. (1994). Classification of Chemical Bonds Based on Topological Analysis of Electron Localization Functions. Nature.

[45] Bartashevich E.V., Tsirelson V.G. (2014). Interplay between Non-covalent Interactions in Complexes and Crystals with Halogen Bonds. Russ. Chem. Rev..

[46] Kuznetsov M.L. (2018). Can Halogen Bond Energy be Reliably Estimated from Electron Density Properties at Bond Critical Point? The Case of the (A)nZ–Y···X− (X, Y = F, Cl, Br) Interactions. Int. J. Quantum Chem..

[47] Dunning T.H. (1989). Gaussian Basis Sets for Use in Correlated Molecular Calculations. I. The Atoms Boron through Neon and Hydrogen. J. Chem. Phys..

[48] Wilson A.K., Woon D.E., Peterson K.A., Dunning T.H. (1999). Gaussian Basis Sets for Use in Correlated Molecular Calculations. IX. The Atoms Gallium through Krypton. J. Chem. Phys..

[49] Zhao Y., Truhlar D.G. (2008). The M06 Suite of Density Functionals for Main Group Thermochemistry, Thermochemical Kinetics, Noncovalent Interactions, Excited States, and Transition Elements: Two New Functionals and Systematic Testing of Four M06-class Functionals and 12 Other Functionals. Theoret. Chem. Acc..

[50] Schmidt M.W., Baldridge K.K., Boatz J.A., Elbert S.T., Gordon M.S., Jensen J.H., Koseki S., Matsunaga N., Nguyen K.A., Su S. (1993). General Atomic and Molecular Electronic Structure System. J. Comput. Chem..

[51] Dovesi R., Saunders V.R., Roetti C., Orlando R., Zicovich-Wilson C.M., Pascale F., Civalleri B., Doll K., Harrison N.M., Bush I.J. (2014). CRYSTAL14 User’s Manual.

[52] Becke A.D. (1993). Density-Functional Thermochemistry. III. The Role of Exact Exchange. J. Chem. Phys..

[53] Lee C., Yang W., Parr R.G. (1988). Development of the Colle-Salvetti Correlation-Energy Formula into a Functional of the Electron Density. Phys. Rev. B Condens. Matter Mater. Phys..

[54] Grimme S. (2006). Semi-empirical GGA-Type Density Functional Constructed with a Long-Range Dispersion Correction. J. Comput. Chem..

[55] Dovesi R., Saunders V.R., Roetti C., Orlando R., Zicovich-Wilson C.M., Pascale F., Civalleri B., Doll K., Harrison N.M., Bush I.J. (2017). CRYSTAL17 User’s Manual.

[56] Gatti C., Casassa S. (2014). Topond14 User’s Manual.

[57] Keith T.A. AIMALL, Version 12.06.03, 2012 Professional. http://aim.tkgristmill.com.

[58] Lu T., Chen F. (2012). Multiwfn: A multifunctional wavefunction analyzer. J. Comput. Chem..

[59] Kostenetskiy P., Semenikhina P. SUSU Supercomputer Resources for Industry and Fundamental Science. Proceedings of the Global Smart Industry Conference (GloSIC).

