# Supplementary material for: Identification of the Tetrel Bonds between Halide Anions and Carbon Atom of Methyl Groups Using Electronic Criterion
Source: Molecules. 2019 Mar 19;24(6):1083. doi: 10.3390/molecules24061083 (PMC6471983; doi:10.3390/molecules24061083)
Supplement: Supplementary file 1 [file molecules-24-01083-s001.pdf]

molecules

MDPI

Type of the Paper Article

Identification of the Tetrel Bonds between Halide Anions and Carbon Atom of Methyl Groups Using Electronic Criterion

Ekaterina Bartashevich [1,*], Yuriy Matveychuk [1] and Vladimir Tsirelson [1,2]

[1] South Ural State University Chelyabinsk, Russia; info@susu.ru
[2] D.I. Mendeleev University of Chemical Technology, Moscow, Russia;
* Correspondence: bartashevichev@susu.ru;

Table S1. The energy characteristics of $Hal^{-}\cdots CH_3$–YR (Hal^{-} = Cl, Br) complexes taken from crystal structures with listed refcodes

Refcode	E_{com} [2]	E_{cat} [2]	E_{Hal} [2]	E_b [2]	ΔE_{BSSE} [3]	E_b* [4]
LONGEB (Y=N)	-1227.466350	-767.095777	-460.257420	-71.052830	-0.001526	-71.895402
TMHYZC (Y=N)	-690.470080	-230.081010	-460.257420	-82.668243	-0.001488	-83.217361
VAPREJ (Y=N)	-633.933164	-173.545474	-460.257420	-81.800417	-0.002269	-82.062672
ZENJAD (Y=O)	-993.217431	-532.876748	-460.257420	-52.282767	-0.001837	-53.577581
GETQIF (Y=N)	-635.155302	-174.768773	-460.257420	-81.072254	-0.001438	-81.495893
FADXIR (Y=N)	-3015.071263	-440.680183	-2574.261952	-81.086284	-0.000161	-81.598635
LILLOH (Y=N)	-2747.932725	-173.545490	-2574.261952	-78.670336	-0.000810	-78.901512
POSTUM02 (Y=N)	-2709.858138	-135.469081	-2574.261952	-79.815774	-0.000104	-80.207947
ZZZGVM01 (Y=N)	-2749.154832	-174.768716	-2574.261952	-77.967743	-0.000772	-78.343999
ZZZUQO03 (Y=N)	-2788.451625	-214.067645	-2574.261952	-76.626898	-0.000749	-77.006447

[1] R – residual fragments of cations in crystals with corresponding refcodes

[2] Notations are same as in Calculations section

[3] The value of BSSE correction $\Delta E_{BSSE} = E_b{}^{*}{}_{BSSE} - E_b{}^{*}$, where $E_b{}^{*}{}_{BSSE}$ – the binding energy between halide anion and cation with non-relaxed structures as in optimized complex with BSSE correction

[4] E_b* – the binding energy between halide anion and cation with non-relaxed structure as in optimized complexes

Table S2. Experimental and calculated tetrel and C–Y bond lengths D (Å), angles $Hal^{-}\cdots$C–Y and electron density (a.u.) at bond critical points for considered crystal and cation structures calculated in CRYSTAL code

Crystal	Bond	Crystal D_{exp}, Θ $(Hal^{-}\cdots C–N)_{exp}$	Crystal D_{calc}, Θ $(Hal^{-}\cdots C–N)_{calc}$	$\rho(r_{bcp})$, crystal	Cation D_{calc}	$\rho(r_{bcp})$, cation
GETQIF	$Cl(3)^{-}\cdots C(2)$	3.4584	3.4260	0.0056	–	–

		169.08	163.91			
	C(2)–N(1)	1.4815	1.4958	0.2441	1.505	0.2368
LONGEB	Cl(1)$^-$···C(4)	3.4251<br>175.28	3.4087<br>166.64	0.0068	–	–
	C(4)–N(2)	1.4722	1.4740	0.2458	1.478	0.2450
VAPREJ	Cl(1)$^-$···C(1)	3.417<br>164.88	3.4385<br>164.49	0.0064	–	–
	C(1)–N(1)	1.466	1.4747	0.2480	1.479	0.2442
TMHYZC	Cl(1)$^-$···C(2)	3.4374<br>174.96	3.4280<br>176.34	0.0062	–	–
	C(2)–N(1)	1.4976	1.5080	0.2406	1.508	0.2413
ZENJAD	Cl(1)$^-$···C(7)	3.5111<br>170.58	3.4644<br>171.72	0.0056	–	–
	C(7)–O(2)	1.4471	1.4468	0.2306	1.451	0.2270
JIBDED01	Br(1)$^-$···C(1)	3.4915<br>173.25	3.4436<br>173.24	0.0070	–	–
	C(1)–N(1)	1.4926	1.5015	0.2490	1.519	0.2261
LILLOH	Br(1)$^-$···C(2)	3.533<br>167.25	3.5664<br>166.86	0.0061	–	–
	C(2)–N(1)	1.474	1.4735	0.2490	1.479	0.2442
FADXIR	Br(1)$^-$···C(6)	3.6014<br>170.87	3.5722<br>173.15	0.0058	–	–
	C(6)–N(1)	1.5025	1.5082	0.2371	1.506	0.2389
POSTUM02	Br(1)$^-$···C(1)	3.7012<br>175.21	3.6667<br>174.15	0.0048	–	–
	C(1)–N(1)	1.4852	1.4928	0.2432	1.509	0.2304
ZZZGVM01	Br(1)$^-$···C(2)	3.742<br>168.65	3.7283<br>169.04	0.0042	–	–
	C(2)–N(1)	1.474	1.4968	0.2437	1.505	0.2368
ZZZUQO03	Br(1)$^-$···C(1)	3.685<br>171.12	3.6819<br>171.11	0.0049	–	–
	C(1)–N(1)	1.487	1.5039	0.2411	1.506	0.2399

Table S3. Bond lengths D(Å), the characteristics of electron density, potential and kinetic energy densities (a.u.), electrostatic potential (a.u.), potential acting on an electron in molecule PAEM at bcp (a.u.) for Hal$^-$···CH_3 and Y–C bonds in complexes and cations calculated in GAMESS code

Refcode	Bond	D in complex	D in cations	$\varrho(r_{bcp})$ in complex	$\varrho(r_{bcp})$ in cation	$v(r_{bcp})$	$g(r_{bcp})$	$ESP(r_{bcp})$	$PAEM(r_{bcp})$
GETQIF	Cl$^-$···C	2.8262		0.019		0.0161	-0.0131	0.0022	-0.4944
	N–C	1.5218	1.4947	0.214	0.235	0.1509	-0.3922	1.2734	
LONGEB	Cl$^-$···C	2.8782		0.017		0.0147	-0.0116	-0.0246	-0.4569
	N–C	1.4930	1.4698	0.221	0.242	0.1801	-0.4520	1.3820	
TMHYZC	Cl$^-$···C	2.8248		0.019		0.0164	-0.0133	0.0068	-0.5004
	N–C	1.5185	1.4975	0.220	0.239	0.1491	-0.3980	1.2583	
VAPREJ	Cl$^-$···C	2.8226		0.019		0.0167	-0.0135	0.0076	-0.5006
	N–C	1.4859	1.4722	0.225	0.241	0.2033	-0.4935	1.4894	
ZENJAD	Cl$^-$···C	2.9268		0.015		0.0125	-0.0098	-0.0771	-0.3956
	O–C	1.4844	1.4422	0.196	0.224	0.2124	-0.4483	1.4698	
FADXIR	Br$^-$···C	2.9855		0.017		0.0134	-0.0110	0.0145	-0.4865

	N–C	1.5220	1.4956	0.216	0.237	0.1459	-0.3859	1.2596	
LILLOH	Br⁻···C	2.9896		0.016		0.0135	-0.0109	0.0126	-0.4808
	N–C	1.4835	1.4721	0.226	0.241	0.2029	-0.4963	1.4942	
POSTUM02	Br⁻···C	2.9802		0.017		0.0135	-0.0109	0.0139	-0.4841
	N–C	1.5259	1.4975	0.209	0.229	0.1531	-0.3874	1.3011	
ZZZGVM01	Br⁻···C	2.9949		0.0165		0.0129	-0.0105	0.0042	-0.4727
	N–C	1.5196	1.4949	0.2155	0.235	0.1505	-0.3941	1.2790	
ZZZUQO03	Br⁻···C	3.0015		0.0162		0.0129	-0.0104	0.0056	-0.4702
	N–C	1.4959	1.4959	0.2187	0.238	0.1443	-0.3881	1.2406	

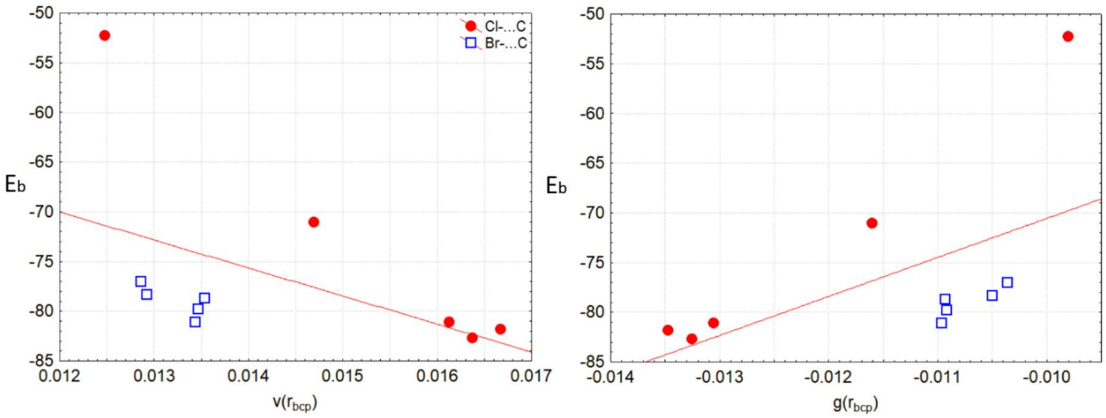

Figure S1. Binding energy (kcal/mol) in complexes vs the potential (a) and kinetic (b) energy density (a.u.) at the bond critical point of tetrel bonds

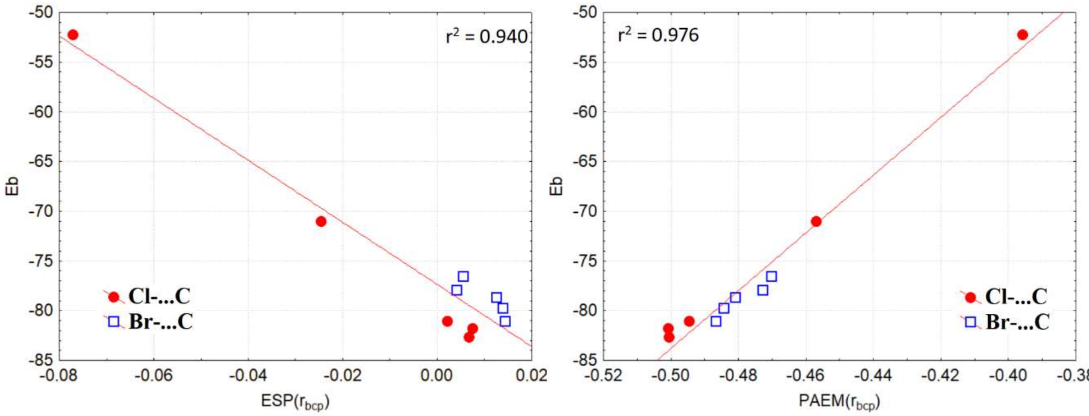

Figure S2. Binding energy (kcal/mol) in complexes vs the electrostatic potential (a.u.) (a) and (b), potential acting on an electron in molecule (a.u.) at the bond critical point of tetrel bon

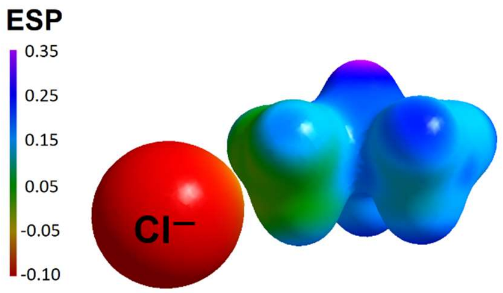

Figure S3. ESP in the trimethylammonium chloride on the isosurface of electron density of 0.02 a.u.
